# Seasonal Variation in Mortality, Medical Care Expenditure and Institutionalization in Older People: Evidence from a Dutch Cohort of Older Health Insurance Clients

**DOI:** 10.1371/journal.pone.0143154

**Published:** 2015-11-16

**Authors:** Herbert Jan Albert Rolden, Jos Hermanus Theodoor Rohling, David van Bodegom, Rudi Gerardus Johannes Westendorp

**Affiliations:** 1 Leyden Academy on Vitality and Ageing, Leiden, The Netherlands; 2 Leiden University Medical Center, Leiden, The Netherlands; 3 Radboud University Medical Center, Nijmegen, The Netherlands; 4 University of Copenhagen, Copenhagen, Denmark; The Ohio State University, UNITED STATES

## Abstract

**Background:**

The mortality rates of older people changes with the seasons. However, it has not been properly investigated whether the seasons affect medical care expenditure (MCE) and institutionalization. Seasonal variation in MCE is plausible, as MCE rises exponentially before death. It is therefore important to investigate the impact of the seasons on MCE both mediated and unmediated by mortality.

**Methods:**

Data on mortality, MCE and institutionalization from people aged 65 and older in a region in the Netherlands from July 2007 through 2010 were retrieved from a regional health care insurer and were linked with data from the Netherlands Institute for Social Research, and Statistics Netherlands (n = 61,495). The Seasonal and Trend decomposition using Loess (STL) method was used to divide mortality rates, MCE, and institutionalization rates into a long-term trend, seasonal variation, and remaining variation. For every season we calculated the 95% confidence interval compared to the long-term trend using Welch’s t-test.

**Results:**

The mortality rates of older people differ significantly between the seasons, and are 21% higher in the winter compared to the summer. MCE rises with 13% from the summer to the winter; this seasonal difference is higher for the non-deceased than for the deceased group (14% vs. 6%). Seasonal variation in mortality is more pronounced in men and people in residential care. Seasonal variation in MCE is more pronounced in women. Institutionalization rates are significantly higher in the winter, but the other seasons show no significant impact.

**Conclusions:**

Seasonal changes affect mortality and the level of MCE of older people; institutionalization rates peak in the winter. Seasonal variation in MCE exists independently from patterns in mortality. Seasonal variation in mortality is similar for both institutionalized and community-dwelling elderly. Policy-makers, epidemiologists and health economists are urged to acknowledge and include the impact of the seasons in future policy and research.

## Introduction

Developed countries are faced with ageing populations and increasing health care expenses. Investigating the determinants of health care expenditure in the older population is therefore of high importance. Many epidemiological studies show that there is seasonal variation in mortality [1–16], but whether medical care expenditure (MCE) also shows seasonal variation has not been formally investigated. Since individual levels of MCE rise steeply prior to death [17–22], it seems only logical that—as a direct result of seasonal variation in mortality—there is seasonal variation in MCE. However, the level of MCE may also change with the seasons due to changes in non-fatal forms of morbidity. It is therefore tempting to study seasonal differences in older people’s health also by focusing on changes in health care utilization and expenditure separately for individuals who died and those who survived.

It has been found that mortality rates in the older population rise during winter time in Europe [8–10, 16], the US [6], and low and middle income countries [5, 12], and New-Zealand [4]. Summers are also associated with higher mortality rates [2, 3, 6, 11]. A very recent multi-country investigation shows that mortality rates gradually increase with colder temperatures, but also show a sudden increase when excessive heat occurs [7]. Many different factors are thought to mediate the association between ambient temperature and morbidity and mortality, such as changes in the risk cardiovascular events [23, 24], susceptibility to infectious diseases [14, 25, 26], and the risk of incurring a hip fracture [27]. The occurrence of more strokes and hip fractures during cold seasons implies that the demand for institutional care is higher during these seasons. Some studies find that the effect of seasonal variation is more prominent in women than men [4, 9, 16]. Socioeconomic differences did not influence the associations [8, 15].

Here, we study the effect of the seasons on MCE and institutionalization (viz., admittance to a care or nursing home). We aim to answer two research questions: (1) is there seasonal variation in mortality rates, medical care expenditure (MCE), and institutionalization rates (admission rates to care and nursing homes) in Dutch people aged 65 years or older?; and (2) does seasonal variation in mortality rates, MCE and/or institutionalization rates differ between subgroups, based on gender, age, vital status (close to death or not), and residential status (institutionalized vs. community-dwelling)? We use the ‘STL decomposition method’ to detect a possible seasonal variation in the different measures. This method divides the data into a long-term trend, seasonal variation around the long-term trend, and ‘remaining’ variation. For the second research question we divide the study population into different subgroups.

## Methods

### The Dutch health care system

Health care in the Netherlands can be separated into two main sectors: medical care and long-term care. Medical care refers to consultations, medication and treatment from general practitioners, medical specialists, dentists, pharmacists, and therapists (such as physiotherapists and psychotherapists). Some forms of instrumental aid and transportation are also provided through the medical care sector. In the studied time period, health care providers bill health care insurers in the form of diagnosis related groups. Medical care is legally arranged through the Health Insurance Act (HIA).

Long-term care in the Netherlands is legally arranged through the Exceptional Medical Expenses Act (EMEA). Entitled to care through the EMEA are people who cannot provide in their basic care needs independently due to a physical, psychogeriatric or psychiatric ailment, or a mental, physical or sensorial handicap. Before someone may receive long-term care through the EMEA, the Center for Indication Setting has to evaluate the client’s health status and issue an official indication. When an indication is set for a client, the actual provision of long-term care is arranged by so-called *care offices*. After an indication is set, the care office appoints a health care provider for a client, or distributes a personal budget. The health care insurer who has the highest share of clients in a region acts as the care office for that region.

Indications for residential care are defined in type and level (hours per week). Since July 1^st^ 2007, residential care is indicated in terms of Care Weight Packages (CWPs). CWPs are pre-defined bundles of care, consisting of different types of long-term care on different levels, complemented with residence. From July 2007 through December 2010, residential care was categorized into ten CWPs: the first four relate to different types of residential care in care homes, the second four to nursing homes, the 9^th^ to rehabilitation, and the 10^th^ to palliative care.

### Ethics statement

After consulting the internal review board (IRB) of the regional health insurer, data on health care expenditure were retrieved from a health insurer. A formal waiver of IRB approval was received from Statistics Netherlands (*Centraal Bureau van de Statistiek*) for data collection. After consulting the IRB of Statistics Netherlands, a single transfer of data from the health insurer to Statistics Netherlands was undertaken over a secure line. A formal waiver was received by the IRB of Statistics Netherlands. After the data transfer, the Statistics Netherlands first removed any personal data. The leading author could then only access the de-identified data in a secured room of Statistics Netherlands. The authors had no access to identifying information. Any output destined for publication was first scrutinized by the IRB of Statistics Netherlands, so no output could be traced back to individuals. No data are publicly available. Data collection and analysis was in full accordance with privacy legislation and protocol.

### Data

With the aim to perform multiple studies on the association between the life situation of older people and their health care expenses, the *Leiden Health care Costs in Old Age* (LHCOA) study was started in 2011. For this study, data on health care expenses of 61,495 people aged 65 and older in a period of 42 months were retrieved from a regional Dutch health insurance company (*Zorg & Zekerheid*) and matched with data on socio-economic characteristics from Statistics Netherlands. Data were collected using the following steps:

1. After consulting the IRB (legal department) of the regional health insurance company, data on MCE were retrieved from the management information system of the health insurance company. Data were collected for the period July 2007 through 2010 for all persons who lived in the regions where the health care insurer acted as the long-term care office, and who reached the age of 65 before 2011. Addresses were linked with data on socio-economic status by postal code, provided by the Netherlands Institute for Social Research (*Sociaal Cultureel Planbureau)*.
2. In accordance with the IRB of the health insurance company, data on long-term care utilization were collected from the EMEA Care Registration system (ECR), an information system which offers an oversight of all the coded messages that are sent between organizations active within the confounds of the EMEA. ECR messages designating the start and end of long-term care provision were used to determine whether a client was institutionalized. Institutionalization rate was defined as the admission rate to care and nursing homes.
3. After consulting the IRB of Statistics Netherlands a single transfer of data from the health insurer to Statistics Netherlands was done over a secure line. CBS staff merged the data using citizen service numbers and dismissed any personal data afterwards. The authors could then only access the de-identified data in a secured room of Statistics Netherlands. The authors had no access to identifying information. Any output destined for publication was first scrutinized by the IRB of Statistics Netherlands, so no output could be traced back to individuals. Data collection and analysis was in full accordance with privacy legislation and protocol. Socio-demographic variables collected at Statistics Netherlands were: age, gender, marital status, and time of death.

The total study population of the LHCOA study (n = 61,495) was used to investigate the association between the seasons and mortality, medical care expenditure (MCE) and institutionalization rate. If an association between seasonal change and mortality as well as MCE is found, it will be unclear whether the association with MCE is mediated by mortality in combination with the high costs of dying, or whether there is a direct association between seasonal changes and MCE. Therefore, the total study population was divided into two groups: subjects who died before 2012 (n = 9,202), and those that survived until 2012 (n = 52,293). If an association between seasonal changes and MCE exists in the survivor group, there is evidence for a direct association, unmediated by mortality rates and the costs of dying.

Besides splitting up the study population into a deceased and non-deceased group, we also separated subgroups on the basis of age, gender, and residential status. We divided the population in community-dwellers and institutionalized subjects to further investigate the link between mortality and outside temperature. Institutionalized subjects were admitted in a care home or nursing home and were therefore predominantly or even exclusively exposed to a constant inside temperature. If there is also seasonal variation in mortality for the institutionalized, it is plausible that seasonal variation is caused by other factors than temperature alone.

### Statistical analysis

Seasons represent three-monthly periods. In the Netherlands, the winter season officially starts on December 21^st^ and ends on March 20^th^. Therefore, winter data were defined as January, February and March. Spring, summer and autumn were defined using the subsequent three monthly periods.

The Seasonal and Trend decomposition using Loess (STL) method was used to analyse seasonal variation in mortality risk, MCE, and institutionalization rate. The STL method decomposes longitudinal data into a long-term trend, seasonal variation, and remaining variation that does not stem from the long-term trend or from seasonal variation [28]. The long-term trend in the STL method reflects an array of possible external factors that gradually change over time, such as higher average ages, an increased risk of widowhood, changes in health care policy, and inflation. Our aim is to find whether seasonal changes are a significant component in the actual data. For every season we calculated the 95% confidence interval compared to the long-term trend using Welch’s t-test. The R statistical programming language was used for the STL decomposition.

## Results

Table 1 shows the characteristics of the study population. The study population includes 61,495 subjects aged 65 years and older, with an average individual follow-up of 35.7 months. Socio-demographic characteristics are shown for the first month of follow-up. Also shown is the number of deaths and institutionalizations during follow-up.

**Table 1:** Characteristics of the study population.^a^

	n (%)	
**All subjects**	61,495	
*Gender*		
Men	24,904	(41)
Women	36,591	(59)
*Age*		
65–79	49,438	(80)
80+	12,057	(20)
*Marital status*		
Married	35,082	(57)
Not married	26,413	(43)
*Residential status*		
Community-dwelling	61,130	(99)
Institutionalized	365	(1)
**During follow-up** ^b^		
Deceased	7,040	(11)
Institutionalized	7,223	(12)

^a^ Data on these characteristics refer to the first month of follow-up.

^b^ Average individual follow-up is 35.7 months.

**Fig 1:**
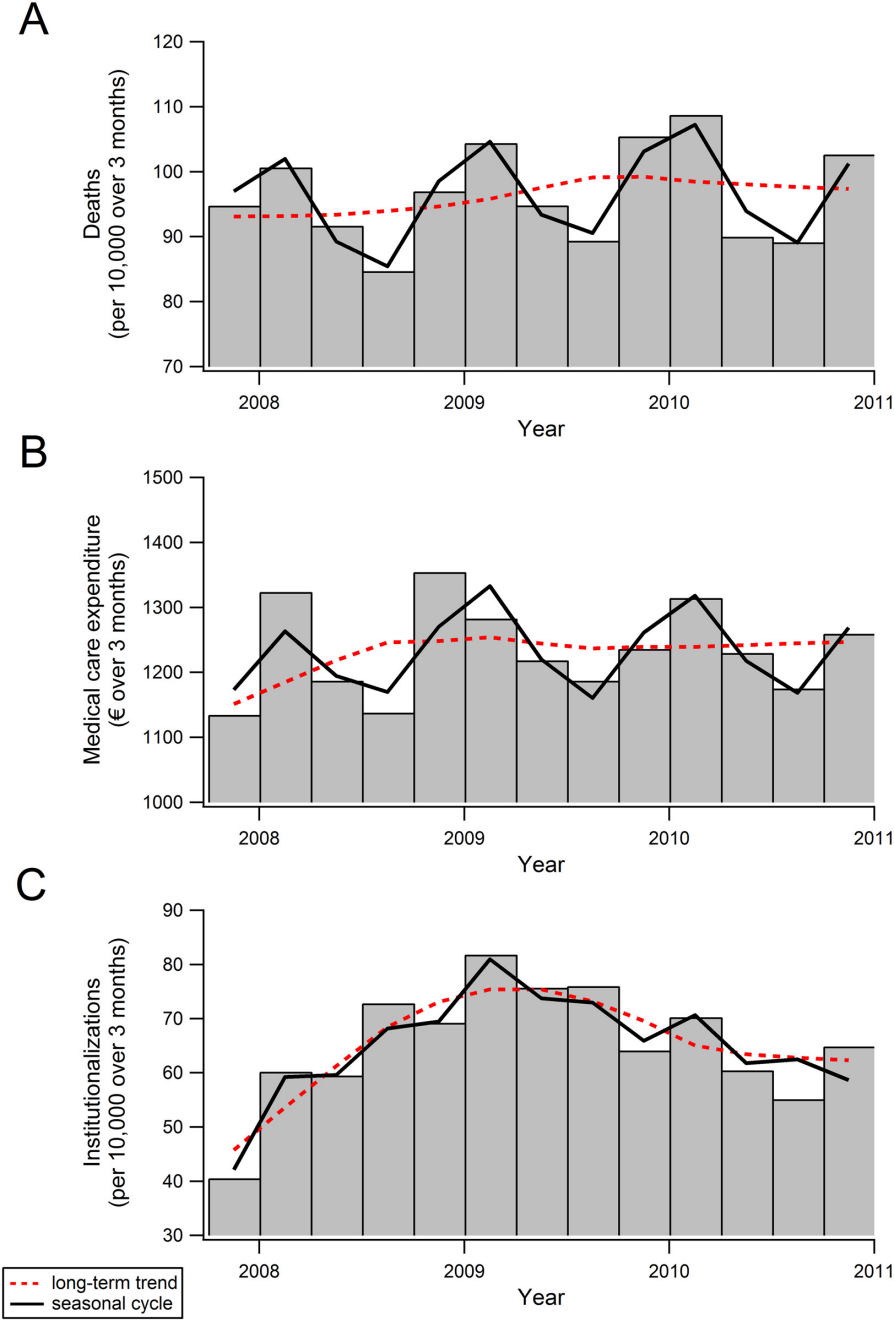
The number of deaths (panel A), level of medical care expenditure (B) and number of institutionalizations (C) in a cohort of Dutch older people. The raw data are decomposed into a long-term trend (red dotted line), and the seasonal variation, or “cycle”, around the long-term trend (black line). Results are from the Seasonal and Trend decomposition using Loess (STL) method.

Fig 1 visualizes the number of deaths, the level of medical care expenditure (MCE), and the number of institutionalizations of the study population, as well as the long-term trends and seasonal cycles around the long-term trends of these variables.

The long-term trend is portrayed with a dotted red line, and the seasonal cycles around the long-term trend are shown with a solid black line. There is a strong long-term trend in institutionalization rate (panel C) between autumn 2007 and autumn 2011. The number of institutionalizations per 10,000 persons for every season rises from 46 in the summer of 2007 to 75 in the winter of 2009. Hereafter, the number slowly decreases towards 62 institutionalizations per 10,000 persons in the autumn of 2010.

**Fig 2:**
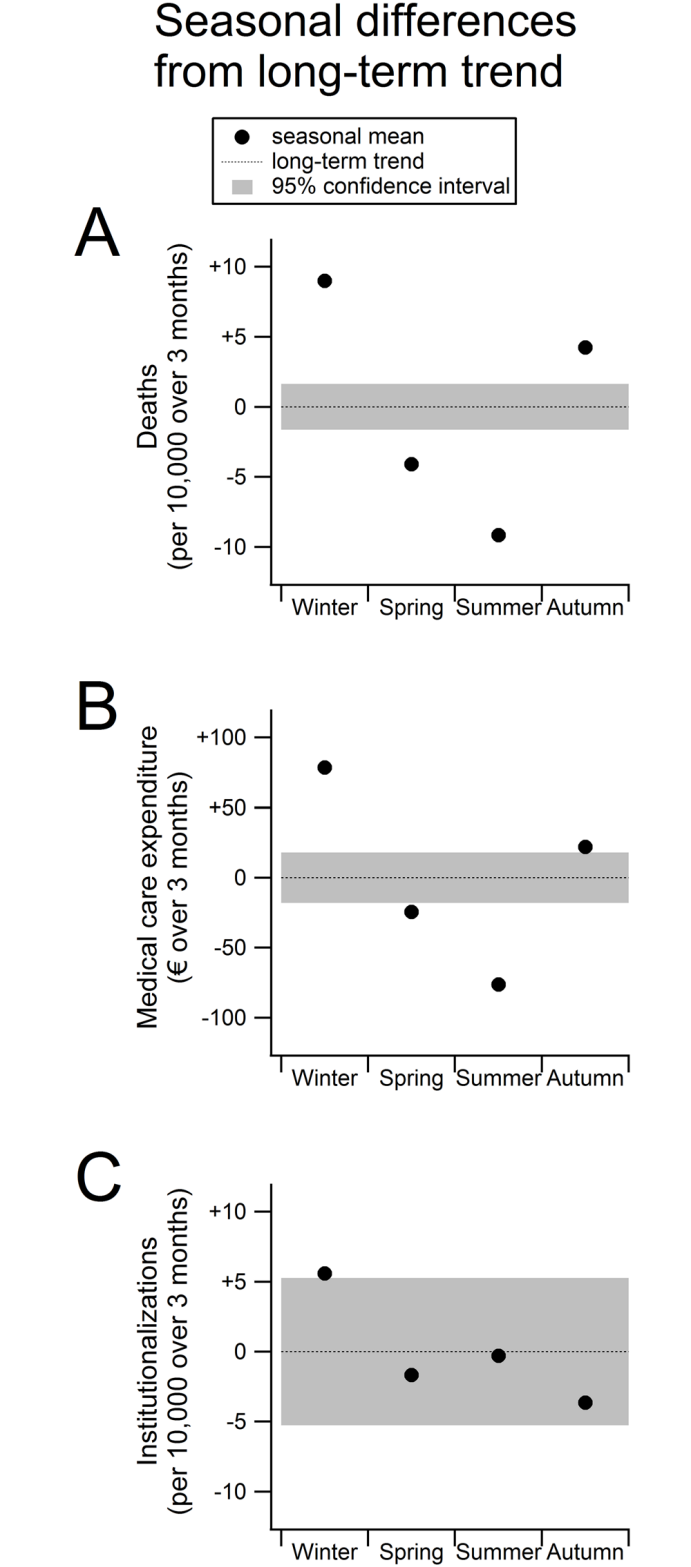
Seasonal variation in mortality (A), medical care expenditure (B) and institutionalizations (C). Shown are the long-term trend and seasonal cycles around the long-term trend according to the Seasonal and Trend decomposition using Loess (STL) method. The dotted line represents the long-term trend, which is set to 0. The colored band around 0 expresses the 95% confidence interval of the long-term trend. The four point estimates in each panel form the mean difference from the long-term trend for each season. When the point estimate of the seasonal component is outside this confidence interval, it is significantly different from the long-term trend.

Fig 2 shows the seasonal variation for mortality rates (panel A), MCE (panel B), and institutionalization rates (panel C). The figure expresses the absolute difference from the long-term average. The average of all years and seasons is set at zero with the corresponding confidence interval as a coloured band. When the point estimate of the seasonal component is outside this confidence interval, it is significantly different from the long-term trend. Visible from Fig 2 is that all seasonal means differ significantly from the long-term trend for mortality and MCE. Institutionalization rates only differ significantly from the long-term average during the winter period.

The average number of deaths (Fig 2, panel A) in every season (all three months) was 96 per 10,000 persons from July 2007 through 2010 according to the long-term trend. On average, there are 9 more deaths per 10,000 population in all three months of the winter period (+9%). Similarly, per 10,000 population there are 4 less deaths in the spring (–4%), 9 less in the summer (–9%), and 4 more in the autumn (+4%). Therefore, the average number of deaths per 10,000 population was 87 in the summer and 105 in the winter. Relatively, the number of deaths in older people is thus 21% higher in the winter than in the summer.

Panel B of Fig 2 shows that MCE is also higher in the autumn and winter than in the spring and summer. Average MCE per person was €1,231 per season (all three months) in the studied time period (based on the long-term trend). Expenditure per person was €79 above this average in the winter (+6%), €24 lower in the spring (–2%), €76 lower in the summer (–6%), and €23 higher in the autumn (+2%). Consequently, seasonal MCE rises with 13% from the summer to the winter (€1,155 vs. €1,310 per person).

The average number of institutionalizations was 65 per 10,000 persons in the study period. Only the institutionalization rate in the winter differs significantly from the long-term trend, when, on average, 6 more older people are institutionalized per 10,000 population. Consequently, there is a rise of 9% in institutionalizations in the winter compared the long-term average, and, since the institutionalization rate in the summer equals the long-term average, it is also 9% higher when comparing the winter to the summer (71 vs. 65 per 10,000 persons). The autumn is characterized by the lowest number of institutionalizations.

In Table 2, the specific data are shown for the mortality rates, MCE, and institutionalization rates for the total study population (described above), as well as for subgroups based on gender, age, residential status and vital status. Visible in Table 2 is that for both the deceased and non-deceased subgroup, MCE is higher in the autumn and winter and lower in the spring and summer. Average MCE was €1,022 per season for individuals surviving, and expenditure was €62 above this average in the winter (+6%), €14 lower in the spring (–1%), €69 lower in the summer (–7%), and €20 higher in the autumn (+2%). Consequently, MCE for the non-deceased group is 14% higher in the winter than in the summer (€1,084 vs. €953). In contrast to all the other subgroups, the lowest level of MCE in the deceased group is not reached in the summer, but in the spring. Seasonal variation is less pronounced in the deceased group: MCE is 6% lower in the summer than in the winter for individuals in their last year before death (€4,385 vs. €4,137), and 7% lower in the spring (€4,385 vs. €4,102).

**Table 2:** Seasonal variation in mortality rates, medical care expenditure and institutionalization rate of an older population (n = 61,495), divided into different subgroups, in the Netherlands from July 2007 through 2010.

	Deaths (per 10,000 over 3 months)					Medical care expenditure (€ over 3 months)					Institutionalizations (per 10,000 over 3 months)				
	*Yearly average*	*Difference from the long-term trend*				*Yearly average*	*Difference from the long-term trend*				*Yearly average*	*Difference from the long-term trend*			
		Winter	Spring	Summer	Autumn		Winter	Spring	Summer	Autumn		Winter	Spring	Summer	Autumn
**All subjects (n = 61,495)**	96	**+9**	**−4**	**−9**	**+4**	1,231	**+79**	**−24**	**−76**	**+22**	65	**+6**	−2	0	−4
*Gender*															
Male (24,904)	117	**+15**	**−10**	**−12**	**+7**	1,328	**+59**	−9	**−72**	**+22**	46	**+5**	**−5**	+1	−1
Female (36,591)	82	**+5**	0	**−7**	**+3**	1,167	**+91**	**−34**	**−79**	+21	78	+6	+1	-1	−5
*Age*															
65–79 (49,438)^a^	55	**+5**	**−3**	**−6**	**+4**	1,157	**+68**	**−20**	**−79**	**+31**	26	**+2**	0	0	**−2**
80+ (12,057)	215	**+21**	**−7**	**−17**	+4	1,446	**+109**	**−36**	**−68**	−5	179	**+15**	−6	−1	−8
*Deceased*															
No (53,391)	-----N/A-----					1,022	**+62**	−14	**−69**	+20	-----N/A-----				
Yes, <1 year (n = 9,202)^b^						4,235	**+150**	**−133**	**−98**	**+80**					
*Residential status*															
Community (n = 53,907)	74	**+7**	**−3**	**−7**	**+3**	-----N/A-----					-----N/A-----				
Institutionalized (n = 7,588)	798	**+93**	−36	**−94**	+37										

**Bold figures** are significantly different from the long-term trend at p<0.05. N/A = Not applicable, or not of interest.

^a^ Here, the number of subjects per age group is defined at baseline; subjects can move from the 65–79 to the 80+ group during follow-up.

^b^ The number of deceased in this table differs from that in Table 1, because Table 1 included only those who died during follow-up, and this table included all subjects who died during follow-up or within one year after follow-up.

There is greater seasonal variation in the mortality of men than that of women: the male mortality rate is 26% higher in the winter than in the summer, and this difference is 16% for women. In contrast, seasonal changes in MCE are more prevalent for women than for men: MCE is 16% higher in the winter than in the summer for women, and this difference is 10% for men. There is not much difference between the two age groups concerning seasonal variation in mortality rates, MCE and institutionalization rates. However, seasonal variations are slightly more concentrated in a winter peak for the older patients, and more dispersed throughout the seasons for younger patients.

There is seasonal variation in mortality rates for both community-dwelling and institutionalized subjects. For community-dwellers mortality rates differ significantly from the long-term trend in all the seasons. The average number of deaths per 10,000 persons was 67 in the summer and 81 in the winter, meaning the number of deaths are 21% higher in the winter compared to the summer. With 27%, this difference between the winter and summer is higher for those who were institutionalized. For them, mortality rates differ significantly from the long-term trend in the winter and summer only.

Institutionalization rates are consistently lowest in the autumn and highest in the winter, regardless of which subgroup is considered. The size of the relative differences between the autumn and the winter are also similar across subgroups: the lowest difference is 13%, found in males and those aged 80 years and older, while the highest difference is 17%, found in those aged younger than 80 years.

## Discussion

We find that older people die more in the winter and autumn than in the spring and summer. Furthermore, their average level of MCE is considerably higher in the winter than in the spring and summer, and their risk of institutionalization peaks in the winter. Seasonal variation is stronger for men than women, but rather similar between age groups, deceased and non-deceased subjects, and institutionalized and community-dwelling elderly.

### Interpretation of results

Previous studies already showed that mortality in the older population is associated with the seasons [1–16], but seasonal variation in MCE is a novel finding. The changes in mortality, MCE and institutionalization suggest that older people’s health is affected by the seasons. It has been found that health is affected by harsh climate conditions, such as cold temperatures [1, 2, 4, 7–10, 12, 13, 15, 16] and heat waves [2, 3, 6, 7, 9, 11–13]. In contrast to some of these studies, we did not find increased mortality rates during the summer. Higher mortality rates in the summer are related to extremely hot temperatures, mainly during heat waves. Extreme hot temperatures are not common in the Netherlands, and, when they occur, usually last only several days.

Different biological pathways could underlie the influence of ambient temperature on older peoples’ health. For example, it has been found that colder temperatures are associated with increased blood pressure [29, 30], higher blood-clotting activity [31], and decreased lung function in COPD patients [32]. In contrast, very warm temperatures can also cause physical problems. For example, the thermoregulatory function and heart rate variability of older people decrease during extremely warm days [33, 34]. We could not measure whether temperature had a strong influence on mortality, MCE and institutionalization as the time frame of our study is too short for this purpose. Also, the data points in our data represent months and quarter years, not days, making the data too crude to investigate this properly. The finding that the mortality rates of institutionalized elderly changes with the seasons suggests that outside temperature may not even play a major role in the seasonal variations found in this study, as the institutionalized elderly are subjected to relatively constant inside temperatures.

Besides ambient temperature, there are many different season-dependent factors that potentially affect population health. First, it is possible that flu underlies seasonal variation in mortality and MCE, as prevalence rates of flu are higher in the winter, and institutionalized people are also—or even more—susceptible to flu compared to those in the community setting [35]. Second, snow and ice may increase the risk of falls and resulting fractures, especially in older people in the community setting [26]. Third, studies have shown that sunlight has a positive effect on health, such as pain after spinal surgery [36], and length of stay and survival after myocardial infarction [37]. Fourth, the level of air pollution shows an association with seasonal changes, and air pollution has an important impact on health outcomes, such as mortality [38], stroke [39], diabetes [40], mental health [41, 42], and even atherosclerosis [43]. Fifth, there is evidence that atmospheric pressure and humidity also affect population health [44, 45]. Sixth, and finally, it is possible that the demand and supply of outpatient care is lower during the summer due to the summer holidays of clinicians and patients, also leading to lower expenditure levels in this season. However, if this would be a major cause of the found seasonal variation in MCE, it would be more plausible that MCE is highest in the autumn, and not in the winter, because outpatient care would peak after the summer holidays.

A higher prevalence of flu in the winter, as well as reduced hours of sunlight, may explain why older people in residential care also show seasonal variation in their mortality rates. Our analysis shows that men suffer more from seasonal changes than women, which stands in contrast to other findings [3, 8, 15]. It is unclear why our results differ from these previous findings, but it is possible that our study population shows discrepancies with those used in previous studies.

There are different explanations for the winter peak in institutionalizations. First, it is possible that climate conditions drive up the demand for long-term care. In this case, an exacerbation of chronic illnesses in the winter could stimulate elderly patients or their family to choose for more intensive long-term care. Second, it is possible that higher mortality rates in the winter drive up the supply of long-term care. In other words, a higher number of deaths results in a higher number of vacancies, and, consequently, a higher rate of institutionalizations if care and nursing homes are functioning at full capacity. Third, although small changes in long-term care policy took place from July 2007 through 2010 –some specific types of home care were abolished—it is implausible that these changes caused a peak in institutionalizations in the beginning of each year.

### Strengths and weaknesses

The existence of seasonal variation in MCE, both close to death as well as independent from impending death, and institutionalization is a novel finding. For the purpose of our analysis, we were able to analyse data on a large study population, which could be separated into several subpopulations, and we used an advanced statistical method to disentangle seasonal variation from a long-term trend and other variation. However, the time period over which we could perform our analysis was rather short (three and a half years). Because, in addition, the data pertained to monthly or quarterly averages, we could not assess the proportional impact of flu, snow and ice, air pollution, sunlight, ambient temperatures, or other climate conditions on mortality, MCE and institutionalization. Another issue pertains to the representativeness of the study population. The study population consists of clients from one health insurer within specific regions of the Netherlands. Although the study population is quite comparable to the Dutch population aged 65 and older in terms of gender and marital status, the number of people aged 80 and older in the study population was relatively low (20% vs. 26.5%) [46]. We could not ascertain how representative our study population was in terms of other characteristics.

### Implications and future research

We performed this study for three reasons: (1) to make policy-makers aware of the impact the seasons can have on the health of older people (if any), as well as the collective health care budget; (2) to show epidemiologists and health economists if and how much the seasons affect MCE and institutionalization rates; and (3) to find out if specific patients groups are more at risk during certain seasons—in this case policy-makers and clinicians will know which patient groups will most likely benefit from targeted interventions.

If the findings from our analysis of the older population in this region of the Netherlands are translated to the entire older Dutch population in 2012 –with a size of ±2.7 million—they would imply that there were 6,880 more deaths amongst older people in the Netherlands in the autumn and winter than in the spring and summer [46]. In addition, MCE of older people were 630 million Euros higher in the cold than the warm seasons in 2012. For epidemiologists and health economists, it is imperative to acknowledge the possible existence of seasonal variation in their data, and include variables representing seasonal variation when performing longitudinal or time series analyses. Policy-makers and health care insurers should be aware of the seasonal differences in MCE and institutionalization to be able to effectively allocate resources in the budgets under their control.

Furthermore, seasonal variation in mortality rates and medical care expenditure could warrant policy changes that could benefit older people and/or decrease the expenditure levels. The finding that institutionalized elderly have a more pronounced seasonal variation in mortality, show that these people may benefit from programmes targeting health care workers’ vaccination or hygiene, improving sunlight exposure or vitamin D status, or promoting better climate control in residential care facilities. To prevent increased levels of MCE or deaths of community-dwellers in the colder seasons, it is of primary importance that we understand which climate conditions impact their health, and through which biological pathways.

Further investigations into seasonal changes in health, MCE and mortality should preferably use daily data over a long period in time, and include many different variables, such as ambient temperature, flu prevalence, sunlight hours, air pollution, atmospheric pressure, and humidity. Furthermore, including data on the types of illness or causes of death could disclose which biological pathways are mainly associated with seasonal changes in health and expenditure.

## Conclusions

Seasonal changes affect the mortality risk and level of medical care expenditure of older people; both outcomes are highest in the winter and lowest in the summer. Institutionalization rates peak in the winter. Seasonal variation is stronger for men than women, but similar between age groups. Changes in outside temperature cannot solely explain the seasonal variation in mortality because seasonal variation in mortality is similar for institutionalized and community-dwelling elderly. Also, seasonal variation in mortality cannot solely explain the variation in medical care expenditure, because the seasonal variation in medical care expenditure is present in the deceased and non-deceased population. Policy-makers, epidemiologists and health economists are urged to acknowledge and include the impact of the seasons in future policy and research. In a time where many countries are faced with population ageing, it is imperative that we gain insight into the causes of seasonal-dependent health deterioration as well as the best clinical means and policy measures to improve the health of older people.

## References

[1] Analitis A, Katsouyanni K, Biggeri A, Baccini M, Forsberg B, Bisanti L, et al Effects of cold weather on mortality: Results from 15 European cities within the PHEWE Project. Am J Epidemiol 2008; 168: 1397–1408. 10.1093/aje/kwn266 18952849

[2] Anderson BG, Bell ML. Weather-related mortality: How heat, cold, and heat waves affect mortality in the United States. Epidemiol 2009; 20: 205–213. 10.1097/EDE.0b013e318190ee08 PMC3366558 19194300

[3] Basu R, Samet JM. Relation between elevated ambient temperature and mortality: A review of the epidemiologic evidence. Epidemiologic Rev 2002; 24: 190–202. 10.1093/epirev/mxf007 12762092

[4] Davie GS, Baker MG, Hales S, Carlin JB. Trends and determinants of excess winter mortality in New Zealand: 1980 to 2000. BMC Public Health 2007; 7: 263 17892590 10.1186/1471-2458-7-263 PMC2174476

[5] Engelaer FM, van Bodegom D, Mangione JN, Eriksson UK, Westendorp RG. Seasonal variation in child and old-age mortality in rural Ghana. Trans R Soc Trop Med Hyg 2014; 108: 147–153. 10.1093/trstmh/tru007 24473476

[6] Fouillet A, Rey G, Laurent F, Pavillon G, Bellec S, Guihenneuc-Jouyaux C, et al Excess mortality related to the August 2003 heat wave in France. Int Arch Occup Environ Health 2006; 80: 16–24. 16523319 10.1007/s00420-006-0089-4 PMC1950160

[7] Gasparrini A, Guo Y, Hashizume M, Lavigne E, Zanobetti A, Schwartz J, et al Mortality risk attributable to high and low ambient temperature: a multicountry observational study. Lancet 2015; 386: 369–75. 10.1016/S0140-6736(14)62114-0 26003380 PMC4521077

[8] Gemmell I, McLoone P, Boddy FA, Dickinson GJ, Watt GCM. Seasonal variation in mortality in Scotland. Int J Epidemiol 2000; 29: 274–279. 10817125 10.1093/ije/29.2.274

[9] Hajat S, Kovats RS, Lachowycz K. Heat-related and cold-related deaths in England and Wales: Who is at risk? Occup Environ Med 2007; 64: 93–100. 16990293 10.1136/oem.2006.029017 PMC2078436

[10] Healy JD. Excess winter mortality in Europe: A cross country analysis identifying key risk factors. J Epidemiol Community Health 2003; 57: 784–789. 14573581 10.1136/jech.57.10.784 PMC1732295

[11] Johnson H, Kovats RS, McGregor G, Stedman J, Gibbs M, Walton H, et al The impact of the 2003 heat wave on mortality and hospital admissions in England. Health Stat Quart 2005; 25: 6–11. 15804164

[12] McMichael AJ, Wilkins P, Kovats RS, Pattenden S, Hajat S, Armstrong B, et al International study of temperature, heat and urban mortality: The ‘ISOTHURM’ project. Int J Epidemiol 2008; 37: 1121–1131. 10.1093/ije/dyn086 18522981

[13] Medina-Ramón M., Schwartz J. Temperature, temperature extremes, and mortality: A study of acclimatisation and effect modification on 50 US cities. Occup Environ Med 2007; 64: 827–833. 17600037 10.1136/oem.2007.033175 PMC2095353

[14] Reichert TA, Simonsen L, Sharma A, Pardo SA, Fedson DS, Miller MA. Influenza and the winter increase in mortality in the United States, 1959–1999. Am J Epidemiol 2004; 160: 492–502. 15321847 10.1093/aje/kwh227

[15] Van Rossum CTM, Shipley MJ, Hemingway H, Grobbee DE, Mackenbach JP, Marmot MG. Seasonal variation in cause-specific mortality: Are there high risk groups? 25-year follow-up of civil servants from the first Whitehall study. Int J Epidemiol 2001; 30: 1109–1116. 11689530 10.1093/ije/30.5.1109

[16] Wilkinson P, Pattenden S, Armstrong B, Fletcher A, Kovats RS, Mangtani P, et al Vulnerability to winter mortality in elderly people in Britain: Population based study. BMJ 2004; 329: 647 15315961 10.1136/bmj.38167.589907.55 PMC517639

[17] Forma L, Rissanen P, Aaltonen M, Raitanen J, Jylhä M. Age and closeness of death as determinants of health and social care utilization: a case control study. Eur J Public Health 2009; 19: 313–318. 10.1093/eurpub/ckp028 19286838

[18] Seshamani M, Gray AM. Ageing and health-care expenditure: the red herring argument revisited. Health Econ 2004; 13: 303–314. 15067669 10.1002/hec.826

[19] Stearns SC, Norton EC. Time to include time to death? The future of health care expenditure predictions. Health Econ 2004; 13: 315–327. 15067670 10.1002/hec.831

[20] Yang Z, Norton EC, Stearns SC. Longevity and health care expenditures: The real reasons older people spend more. J Gerontol B Psychol Sci Soc Sci 2003; 58: S2–S10. 12496303 10.1093/geronb/58.1.s2

[21] Zweifel P, Felder S, Meiers M. Ageing of population and health care expenditure: A red herring? Health Econ 1999; 8: 485–496. 10544314 10.1002/(sici)1099-1050(199909)8:6<485::aid-hec461>3.0.co;2-4

[22] Rolden HJA, van Bodegom D, Westendorp RGJ. Variation in the costs of dying and the role of different health services, socio-demographic characteristics, and preceding health care expenses. Soc Sci Med 2014; 120: 110–117. 25238558 10.1016/j.socscimed.2014.09.020

[23] Braga ALF, Zanobetti A, Schwartz J. The effect of weather on respiratory and cardiovascular deaths in 12 U.S. cities. Environ Health Perspect 2002; 110: 859–63. 10.1289/ehp.02110859 PMC1240983 12204818

[24] Xu B, Liu H, Su N, Kong G, Bao X, Li J, et al Association between winter season and risk of death from cardiovascular diseases: a study in more than half a million inpatients in Beijing, China. BMC Cardiovasc Disord 2013; 13: 93 10.1186/1471-2261-13-93 24172216 PMC3840603

[25] Fleming DM, Cross KW, Crombie DL, Lancashire RJ. Respiratory illness and mortality in England and Wales. A study of the relationships between weekly data for the incidence of respiratory disease presenting to general practitioners, and registered deaths. Eur J Epidemiol 1993; 9: 571–6. 8150058 10.1007/BF00211429

[26] Moshkowitz M, Konikoff FM, Arber N, Peled Y, Santo M, Bujanover Y, et al Seasonal variation in the frequency of Helicobacter pylori infection: a possible cause of the seasonal occurrence of peptic ulcer disease. Am J Gastroenterol 1994; 89: 731–3. 8172147

[27] Román Ortiz C, Tenías JM, Estarlich M, Ballester F. Systematic review of the association between climate and hip fractures. Int J Biometeorol 2014; 12 13: 10.1007/s00484-014-0945-y 25504270

[28] Cleveland RB, Cleveland WS, McRae JE, Terpenning I. STL: A seasonal-trend decomposition procedure based on Loess. J Off Stat 1990; 6: 3–73.

[29] Brennan PJ, Greenberg G, Miall WE, Thompson SG. Seasonal variation in arterial blood pressure. BMJ 1982; 285: 919–23. 6811068 10.1136/bmj.285.6346.919 PMC1499985

[30] Woodhouse PR, Khaw KT, Plummer M. Seasonal variation of blood pressure and its relationship to ambient temperature in an elderly population. J Hypertens 1993; 11: 1267–74. 8301109

[31] Woodhouse PR, Khaw KT, Plummer M, Foley A, Meade TW. Seasonal variations of plasma fibrogen and factor VII activity in the elderly: winter infections and death from cardiovascular disease. Lancet 1994; 343: 435–9. 7508540 10.1016/s0140-6736(94)92689-1

[32] Donaldson GC, Seemungal T, Jeffries DJ, Wedzicha JA. Effect of temperature on lung function and symptoms in chronic obstructive pulmonary disease. Eur Respir J 1999; 13: 844–9. 10362051 10.1034/j.1399-3003.1999.13d25.x

[33] Kenny HP, Yardley J, Brown C, Sigal RJ, Jay O. Heat stress in older individuals and patients with common chronic diseases. CMAJ 2010; 182: 1053–60. 10.1503/cmaj.081050 19703915 PMC2900329

[34] Ren C, O’Neill MS, Park SK, Sparrow D, Volkonas P, Schwartz J. Ambient temperature, air pollution, and heart rate variability in an ageing population. Am J Epidemiol 2011; 173: 1013–21. 10.1093/aje/kwq477 21385834 PMC3121221

[35] Nicholson KG, Wood JM, Zambon M. Influenza. Lancet 2003; 362: 1733–45. 14643124 10.1016/S0140-6736(03)14854-4 PMC7112395

[36] Walch JM, Rabin BS, Day R, Williams JN, Choi K, Kang JD. The effect of sunlight on postoperative analgesic medication use: a prospective study of patients undergoing spinal surgery. Psychosomatic Med 2005; 67: 156–163. 10.1097/01.psy.0000149258.42508.70 15673638

[37] Beauchemin KM, Hays P. Dying in the dark: sunshine, gender and outcomes in myocardial infarction. J Royal Soc Med 1998; 91: 352–354. 10.1177/014107689809100703 PMC1296806 9771492

[38] Peng RD, Dominici F, Pastor-Barriuso R, Zeger SL, Samet JM. Seasonal analyses of air pollution and mortality in 100 US cities. Am J Epidemiol 2005; 161: 585–594. 15746475 10.1093/aje/kwi075

[39] Shah AS, Lee KK, McAllister DA, Hunter AM, Nair H, Whitely W, et al Short term exposure to air pollution and stroke: systematic review and meta-analysis. BMJ 2015; 350: h1295 10.1136/bmj.h1295 25810496 PMC4373601

[40] Eze IC, Hemkens LG, Bucher HC, Hoffmann B, Schindler C, Künzli N, et al Association between ambient air pollution and diabetes mellitus in Europe and North America: systematic review and meta-analysis. Environ Health Perspect 2015; 123: 381–9. 10.1289/ehp.1307823 25625876 PMC4421762

[41] Bakian AV, Huber RS, Coon H, et al Acute air pollution exposure and risk of suicide completion. Am J Epidemiol 2015; 181: 295–303. 10.1093/aje/kwu341 25673816 PMC4339389

[42] Power MC, Kioumourtzoglou M-A, Hart JE, et al The relation between past exposure to fine particulate air pollution and prevalent anxiety: observational cohort study. BMJ 2015; 350: h1111 10.1136/bmj.h1111 25810495 PMC4373600

[43] Adar SD, Sheppard L, Vedal S, et al Fine particulate air pollution and the progression of carotid intima-medial thickness: a prospective cohort study from the multi-ethnic study of atherosclerosis and air pollution. PLoS Med 2013; 10: e1001430 10.1371/journal.pmed.1001430 23637576 PMC3637008

[44] Danet S, Richard F, Montaye M, Beauchant S, Lemaire B, Graux C, et al Unhealthy effects of atmospheric temperature and pressure on the occurrence of myocardial infarction and coronary deaths: A 10-years survey: The Lille-World Health Organization MONICA project (Monitoring trends and determinants in cardiovascular disease). Circulation 1999; 100: e1–e7. 10393689 10.1161/01.cir.100.1.e1

[45] Ou CQ, Yang J, Ou QQ, Liu HZ, Lin GZ, Chen PY, et al The impact of relative humidity and atmospheric pressure on mortality in Guangzhou, China. Biomed Environ Sci 2014; 27: 917–25. 10.3967/bes2014.132 25484008

[46] Central Bureau of Statistics. Statline. http://statline.cns.nl/Statweb/. Accessed February 15^th^ 2015.

